# RANTES gene polymorphisms and risk of pediatric asthma: A meta-analysis

**DOI:** 10.3892/etm.2012.684

**Published:** 2012-08-28

**Authors:** YAN-MING LU, LAN-FANG CAO, YA-QIN LI, CHEN LI

**Affiliations:** Department of Pediatrics, Renji Hospital Affiliated to Medical School, Shanghai Jiaotong University, Shanghai 200001, P.R. China

**Keywords:** RANTES, pediatric asthma, meta-analysis, polymorphism

## Abstract

Numerous studies have evaluated the association between regulated upon activation, normal T cells expressed and secreted (RANTES) gene polymorphisms (−403G/A and −28C/G) and risk of pediatric asthma. However, the results have been inconsistent. A meta-analysis of the association between RANTES gene polymorphisms and pediatric asthma risk was performed in the current study. A search for published literature was conducted in the Google Scholar, PubMed and the CNKI databases (January 2000 to April 2012) and seven studies were retrieved. The associations between RANTES gene polymorphisms and pediatric asthma risk were estimated by pooled odds ratio (OR) and 95% confidence interval (CI) using a fixed- or random-effects model. Meta-analysis results revealed no significant association between the −403G/A polymorphism and risk of pediatric asthma. In the subgroup analysis by ethnicity, no association was identified between the −403G/A polymorphism and pediatric asthma risk in Caucasian and Asian populations. In the −28C/G group, the meta-analysis indicated a significant association between the −28C/G polymorphism and pediatric asthma susceptibility among the total population (recessive model: OR, 1.34; 95% CI, 1.04–1.72). However, when the subgroup analysis was performed by ethnicity, no significant associations were identified in Asians and Europeans. This result suggests that the −28C/G polymorphism may not be associated with pediatric asthma risk, while the observed increase in the risk of pediatric asthma may be due to racial differences. Additional large-scale studies are required to provide conclusive evidence on the effects of RANTES gene polymorphisms on the risk of pediatric asthma.

## Introduction

Asthma is a chronic inflammatory disease of the respiratory airways leading to episodes of wheezing, shortness of breath, chest tightness and coughing (1). Approximately 300 million individuals are affected by asthma globally, including 10 million children (2). The pathogenesis of asthma is extremely complex and not fully understood. The major risk factors for the development and persistence of asthma are allergic disease, reduced lung function and viral and bacterial infections (3–5). In addition, variants in over 40 genes have been associated with asthma (6). Parental asthma is a strong predictor of childhood asthma, suggesting a strong genetic basis of pediatric asthma (7).

The regulated upon activation, normal T cells expressed and secreted (RANTES) protein is one of the most extensively studied chemokines in allergic and infectious diseases (8). RANTES is likely to be significant in airway inflammation, since blocking antibodies to RANTES reportedly inhibit airway inflammation in a murine model of allergic airway disease (8). Two polymorphisms in the RANTES promoter region (−28 C/G and −403 G/A) have been demonstrated to affect the transcription of the RANTES gene and exacerbate asthma severity (9,10). However, the published results have been controversial. To aid the clarification of the inconsistent findings, with the publication of several more recent studies, this meta-analysis of RANTES gene polymorphisms (−403G/A and −28C/G) and the risk of pediatric asthma was conducted.

## Materials and methods

### Selection of studies

The data were independently gathered in duplicate by two investigators on the basis of a standard protocol (Y.M.L. and L.F.C.). Discrepancies between the investigators were settled by discussion until consensus was reached. Literature databases were searched, including PubMed, Google Scholar and the China National Knowledge Infrastructure (CNKI) databases. The following key words and subject terms were searched: ‘RANTES’, ‘−403G/A’, ‘−28C/G’, ‘pediatric asthma’ and ‘gene polymorphism’. The reference lists of retrieved reviews and articles were hand-searched. The search was without restriction on language and was conducted on human subjects. The literature search was updated on 30 May 2012.

### Selection criteria

The titles and abstracts of all citations identified by the literature search were reviewed. Selection criteria were then applied to all potentially relevant studies. The selection criteria for inclusion in the meta-analysis were: i) case-control studies conducted to evaluate the association between RANTES gene polymorphisms (−403G/A and −28C/G) and pediatric asthma risk; ii) sufficient genotype data were presented to calculate the odds ratios (ORs) and 95% confidence intervals (CIs); iii) the study should clearly describe pediatric asthma diagnoses and the sources of cases and controls. Studies were excluded when: i) they were non-case-control studies that evaluated the association between RANTES gene polymorphisms and pediatric asthma risk; ii) they were case reports, letters, reviews or editorial articles; iii) they were studies based on incomplete raw data and no usable data were reported; iv) they contained duplicate data.

### Data extraction

The following characteristics were collected from each study: first author, year of publication, region of the first or corresponding author, ethnicity, number of cases and controls, number of genotypes and evidence of Hardy-Weinberg equilibrium (HWE; Table I). Ethnicities were classified as Asian or Caucasian. If original genotype frequency data were unavailable in the relevant articles, an email was sent to the corresponding author for additional data. For conflicting evaluations, an agreement was reached following a discussion.

### Statistical analysis

A meta-analysis was performed using the STATA package version 12.0 (Stata Corporation, College Station, TX, USA). The OR corresponding to the 95% CI was used to assess the intensity of the association between RANTES gene polymorphisms (−403G/A and −28C/G) and pediatric asthma under a homozygote comparison (AA vs. aa), a heterozygote comparison (AA vs. Aa), a dominant model and a recessive mode between groups. In the current study, the dominant model was defined as Aa+aa vs. AA, where ‘A’ and ‘a’ are major and minor alleles, respectively, and the recessive model as aa vs. AA+Aa. The distribution of genotypes in the included studies was tested for HWE using the χ^2^ test. The effect of heterogeneity by the Q-test and I^2^ test was also quantified. I2 ranges between 0 and 100% and I^2^ values of 25, 50 and 75% were defined as low, moderate and high estimates, respectively. When a significant Q-test (P<0.10) or I^2^>50% indicated heterogeneity across studies, the random-effects model was used for meta-analysis, otherwise the fixed-effects model was calculated. Sensitivity analysis was performed to assess the stability of the results by excluding the study by Sohn *et al* (16) with genotype distributions not in HWE. Begg’s test was used to provide evidence of publication bias, shown as a funnel plot. P<0.05 was considered to indicate a statistically significant result.

## Results

### Characteristics of studies

**Figure 1:**
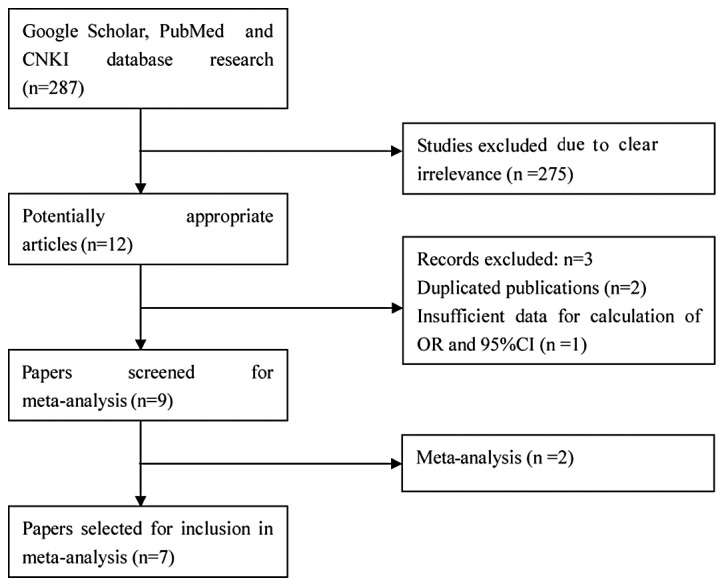
Flow diagram of study searching and selection process.

There were 287 studies relevant to the search words. Through screening the title and reading the abstract and the entire article, 7 eligible articles were selected for this meta-analysis (Fig. 1). All the studies were case-control studies that evaluated the association between RANTES gene polymorphisms and pediatric asthma risk (11–17). For −28C/G, the sample population in the Europe group was inadequate and ethnicity-specific meta-analysis was not conducted on Caucasians. The HWE test was performed on the genotype distribution of the controls in all included studies; all of them were in HWE except that in the study by Sohn *et al* (16). The main characteristics of the included studies are listed in Table I.

### Results of meta-analysis

**Figure 2:**
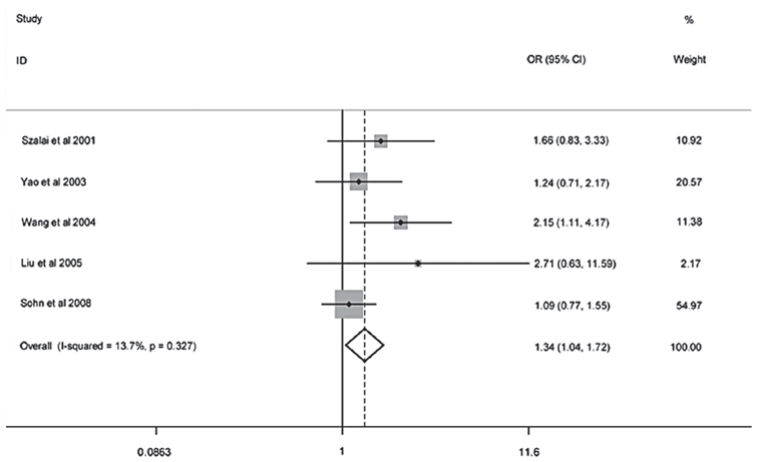
Meta-analysis of the correlation between the −28C/G polymorphism and pediatric asthma risk using a recessive model.

A summary of the meta-analysis findings of the association between RANTES gene polymorphisms (−403G/A and −28C/G) and pediatric asthma risk is shown in Table II. Meta-analysis results showed that there was no association between the −403G/A polymorphism and the risk of pediatric asthma, the meta-analysis indicated that the −28C/G polymorphism was associated with an increased risk of pediatric asthma in the general population (recessive model: OR, 1.34; 95% CI, 1.04–1.72). On the basis of the potential overestimation of the true effect of the polymorphism on the pediatric asthma risk, these studies were stratified according to ethnicity. In the stratified analysis, no significant associations were identified in Asian and European populations. Sensitivity analysis was performed to assess the stability of the results by excluding one study by Sohn *et al* (16) with genotype distributions not in HWE and the exclusion of any single study did not alter the significance of the final decision, suggesting that the outcomes were robust. The funnel plot and Begg’s test were used to assess the publication bias. There was no evidence of publication bias visually from the funnel plot (Fig. 2, Table II). The difference was not significant in Begg’s test (all P>0.05), suggesting that the publication bias was low in the present meta-analysis.

## Discussion

Asthma is the most common chronic childhood disease (18). The pathogenesis of asthma is extremely complex and not fully understood, although the correlation of a variety of genetic loci and multiple environmental factors have been suggested as significant determinants of this disease (19,20). A number of research studies have evaluated the association of RANTES gene polymorphisms (−403G/A and −28C/G) and pediatric asthma risk, but the results are controversial, making it difficult to speculate the true correlation between gene polymorphisms and pediatric asthma. The studies included were conducted in various populations, sample sizes were relatively small and various criteria were used for the phenotype definition (21).

To the best of our knowledge, this is the first meta-analysis to consider RANTES gene polymorphisms and pediatric asthma. This meta-analysis quantitatively assessed the association between RANTES gene polymorphisms (−403G/A and −28C/G) and susceptibility to pediatric asthma. Additonally, the results of our meta-analysis did not show any significant association between −403G/A polymorphism and pediatric asthma risk. As for −28C/G, the meta-analysis indicated a significant association between −28C/G polymorphism and pediatric asthma susceptibility among the total population (recessive model: OR, 1.34; 95% CI, 1.04–1.72). However, when the subgroup analyses were performed by ethnicity, no significant associations were identified in Asian and European populations. This result suggests that the −28C/G polymorphism may not be associated with pediatric asthma risk and the observed increase in the risk of pediatric asthma may be due to a bias of racial differences. Sensitivity analysis was performed to assess the stability of the results by excluding the study by Sohn *et al* (16) with genotype distributions that were not in HWE, suggesting that the results of the meta-analysis were reliable. No evidence revealed publication bias in this meta-analysis for the association between RANTES gene polymorphisms and susceptibility to pediatric asthma.

There were certain limitations to this meta-analysis. First, the linkage disequilibrium of −403G/A and −28C/G of the RANTES gene may synergistically increase the risk of asthma (22). There was insufficient individual information on genotypes of the RANTES −403G/A and −28C/G polymorphisms and combined analysis of linkage disequilibrium was therefore not performed. Consequently, more studies with larger sample sizes and providing more detailed information are required. Second, the effect of gene-gene and gene-environment interactions was not addressed in this meta-analysis. Third, the significance between study-heterogeneity was observed. Although the random-effect model was used to pool ORs, it may have affected the precision of results.

In conclusion, these results suggest that RANTES gene polymorphisms (−403G/A and −28C/G) may not be involved with the susceptibility of pediatric asthma. Owing to the previously mentioned limitations, the findings should be verified by further research in the near future.

## Figures and Tables

**Table I:** Characteristics of the included studies for meta-analysis.

A, −403G/A polymorphism											

First author (Refs.)	Year	Area	Ethnicity	Cases/controls	Case genotypes			Control genotypes			HWE test
					GG	GA	AA	GG	GA	AA	

Szalai (11)	2001	Hungary	Caucasian	160/303	122	32	6	211	84	8	0.92
Yao (12)	2003	China	Asian	182/107	98	65	19	60	41	6	0.77
Liu (13)	2005	China	Asian	32/32	17	13	2	16	14	2	0.64
Leung (14)	2005	China	Asian	129/66	60	53	16	37	21	8	0.09
Tölgyesi (15)	2006	Hungary	Caucasian	144/174	107	34	3	131	40	3	0.98
Sohn (16)	2008	Korea	Asian	326/253	109	146	71	97	107	49	0.05

B, −28C/G polymorphism											

First author (Refs.)	Year	Area	Ethnicity	Cases/controls	Case genotypes			Control genotypes			HWE test
					CC	CG	GG	CC	CG	GG	

Szalai (11)	2001	Hungary	Caucasian	160/303	144	16	0	284	19	0	0.57
Yao (12)	2003	China	Asian	182/107	134	39	9	83	23	1	0.67
Wang (17)	2004	China	Asian	100/90	65	31	4	72	17	1	1.00
Liu (13)	2005	China	Asian	32/32	25	6	1	29	3	0	0.78
Sohn (16)	2008	Korea	Asian	326/253	218	93	15	174	66	13	0.05

HWE, Hardy-Weinberg equilibrium.

**Table II:** Summary ORs and 95% CIs of RANTES gene polymorphisms and pediatric asthma risk.

Subgroup	Genetic model	Sample size		Type of model	Heterogeneity		Association		Publication bias	
		Case	Control		I^2^ (%)	P-value	OR	95% CI	z	P-value
−403G/A										
Overall	AA vs. GG	973	935	Fixed	0.0	0.98	1.34	0.95–1.89	0.00	1.00
	AA vs. GA			Fixed	0.0	0.75	1.18	0.84–1.66	0.00	1.00
	Dominant			Fixed	0.0	0.94	0.81	0.59–1.11	0.00	1.00
	Recessive			Fixed	3.6	0.39	1.06	0.87–1.29	0.00	1.00
Caucasian	AA vs. GG	304	477	Fixed	0.0	0.95	1.27	0.52–3.13	0.00	1.00
	AA vs. GA			Fixed	0.0	0.62	1.66	0.65–4.24	0.00	1.00
	Dominant			Fixed	0.0	0.86	0.73	0.30–1.80	0.00	1.00
	Recessive			Fixed	22.2	0.26	0.84	0.60–1.17	0.00	1.00
Asian	AA vs. GG	669	458	Fixed	0.0	0.87	1.35	0.93–1.96	0.34	1.00
	AA vs. GA			Fixed	0.0	0.61	1.12	0.78–1.62	0.34	1.00
	Dominant			Fixed	0.0	0.75	0.82	0.58–1.15	0.34	1.00
	Recessive			Fixed	0.0	0.81	1.21	0.95–1.54	0.34	1.00
−28C/G										
Overall	GG vs. CC	800	785	Fixed	30.0	0.23	1.53	0.81–2.90	0.24	1.00
	GG vs. CG			Random	82.5	0.00	4.93	0.48–50.53	0.24	1.00
	Dominant			Fixed	26.4	0.25	0.69	0.37–1.29	0.24	1.00
	Recessive			Fixed	13.7	0.33	1.34	1.04–1.72	0.24	1.00
Asian	GG vs. CC	640	482	Fixed	30.0	0.23	1.53	0.81–2.90	1.02	0.31
	GG vs. CG			Random	82.5	0.00	4.93	0.48–5053	1.02	0.31
	Dominant			Fixed	26.4	0.25	0.69	0.37–1.29	1.02	0.31
	Recessive			Fixed	28.6	0.24	1.30	1.00–1.70	1.02	0.31

OR, odds ratio; CI, confidence interval; RANTES, regulated upon activation, normal T cells expressed and secreted.
